# Deep Bisulfite Sequencing of Aberrantly Methylated Loci in a Patient with Multiple Methylation Defects

**DOI:** 10.1371/journal.pone.0076953

**Published:** 2013-10-09

**Authors:** Jasmin Beygo, Ole Ammerpohl, Daniela Gritzan, Melanie Heitmann, Katrin Rademacher, Julia Richter, Almuth Caliebe, Reiner Siebert, Bernhard Horsthemke, Karin Buiting

**Affiliations:** 1 Institut für Humangenetik, Universitätsklinikum Essen, Universität Duisburg-Essen, Essen, Germany; 2 Institute of Human Genetics, Christian-Albrechts-University Kiel & University Hospital Schleswig-Holstein, Campus Kiel, Kiel, Germany; Universität des Saarlandes, Germany

## Abstract

*NLRP7* is a maternal effect gene as maternal mutations in this gene cause recurrent hydatidiform moles, spontaneous abortions and stillbirths, whereas live births are very rare. We have studied a patient with multiple anomalies born to a mother with a heterozygous *NLRP7* mutation. By array-based CpG methylation analysis of blood DNA from the patient, his parents and 18 normal controls on Illumina Infinium HumanMethylation27 BeadChips we found that the patient had methylation changes (delta ß ≥ 0.3) at many imprinted loci as well as at 87 CpGs associated with 85 genes of unknown imprinting status. Using a pseudoproband (permutation) approach, we found methylation changes at only 7-24 CpGs (mean 15; standard deviation 4.84) in the controls. Thus, the number of abberantly methylated CpGs in the patient is more than 14 standard deviations higher. In order to identify novel imprinted genes among the 85 conspicuous genes in the patient, we selected 19 (mainly hypomethylated) genes for deep bisulfite amplicon sequencing on the ROCHE/454 Genome Sequencer in the patient and at least two additional controls. These controls had not been included in the array analysis and were heterozygous for a single nucleotide polymorphism at the test locus, so that allele-specific DNA methylation patterns could be determined. Apart from *FAM50B*, which we proved to be imprinted in blood, we did not observe allele-specific DNA methylation at the other 18 loci. We conclude that the patient does not only have methylation defects at imprinted loci but (at least in blood) also an excess of methylation changes at apparently non-imprinted loci.

## Introduction

DNA methylation is an important epigenetic mark involved in the regulation of gene expression. In mammals, it is mainly present in the context of 5-methylcytosine (5mC) followed by a guanine (CpG dinucleotide) [1]. Regions containing a high degree of CpG dinucleotides are called CpG islands (CGIs). They are often located in the promoter region of genes and are mostly unmethylated [2]. In their methylated state or by the formation of transcriptionally silent heterochromatin they can repress gene transcription [1]. Special cases of DNA methylation are X-inactivation and genomic imprinting. Imprinted loci show a monoallelic, parent-of-origin specific expression most often regulated by a differentially methylated region (DMR) [3]. So far, about 80-90 genes are known to be imprinted in humans, some of them only in specific tissues or at certain times during development [4,5]. In the last years several studies aimed to identify novel imprinted genes in mice and human using different approaches. Luedi and colleagues [6,7] applied a computational method based on DNA sequence characteristics of known imprinted genes for prediction in mice and human. With the development of new, large-scale array and deep sequencing technologies, genome-wide expression and methylation analyses were possible (for review see 8,9). For instance, genotyping microarrays were used with genomic and cDNA to detect differences in allele expression at multiple loci indicative of monoallelic expression and imprinting [10]. Nakabayashi and colleagues [11] combined Illumina Infinium HumanMethylation27 BeadChips (27k arrays) with the use of mosaic genome-wide uniparental disomy samples to identify new imprinted genes for analyses. However, among all predicted novel yet unknown imprinted genes only very few could be shown to be imprinted so far.

Methylation analyses of biparental hydatidiform moles (OMIM #231090) caused by mutations of the maternal effect gene *NLRP7* revealed aberrant methylation at multiple imprinted loci, indicating that maternal *NLRP7* mutations affect establishment and/or maintenance of methylation imprints [12,13]. Homozygous and compound heterozygous mutations in this gene have been identified as a major cause of recurrent biparental hydatidiform mole pregnancies, but also stillbirths and spontaneous abortions were frequently reported, while live births are extremely rare ([14-19] and others). Heterozygous mutation carriers were also described, but reports on reproductive outcomes vary [18,20-22].

Recently, Caliebe et al. (in preparation) identified a family, in which two fetuses and one child of a mother with a heterozygous *NLRP7* mutation (p.A719V) showed altered DNA methylation patterns at many imprinted loci. This family was instrumental in identifying *RB1* as being imprinted [23]. In order to identify novel imprinted genes, we analysed additional candidate genes by deep bisulfite amplicon sequencing. With this technique up to several thousand clonally amplified single sequence reads per sample can be obtained, thus enabling a highly quantitative methylation analysis, which makes it possible to detect even small differences in methylation levels as for example seen in mosaic imprinting defects.

## Results and Discussion

**Figure 1:**
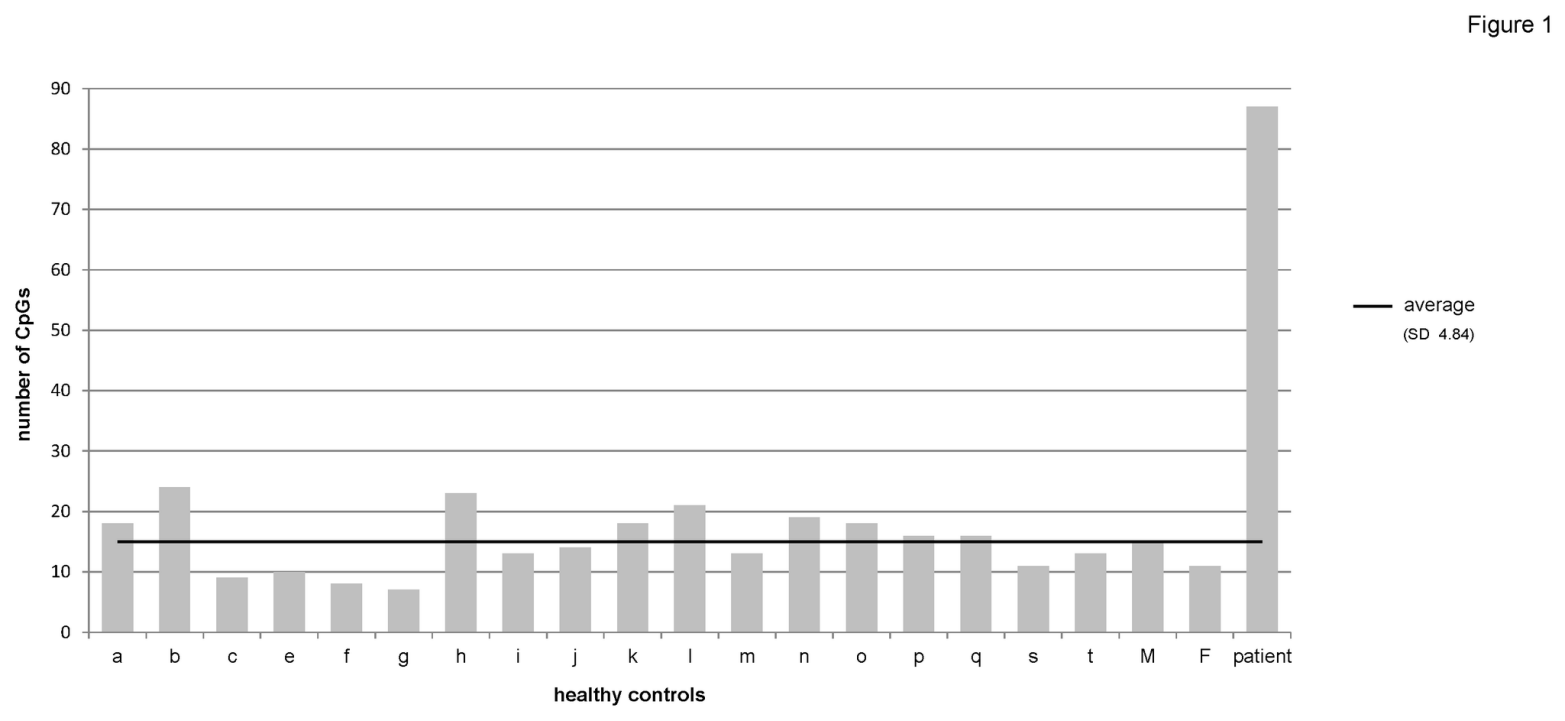
Results of the pseudoproband approach. The diagram shows the number of CpGs in all investigated individuals passing the threshold of delta β ≥ 0.3 when assigned as a pseudoproband. All imprinted genes were excluded. a - t – healthy controls; M – mother of the patient; F – father of the patient; average – average number of CpGs showing a deviation in normal controls and the parents.

We have studied the only affected live-born individual from a family with multiple imprinting defects. To determine the extent of aberrant DNA methylation in the patient and to identify novel imprinted genes, we performed high-throughput methylation profiling of blood DNA from the patient, his parents and 18 normal controls on Illumina Infinium HumanMethylation27 BeadChips (27k arrays), which interrogate 27,578 CpG sites. Based on the raw data (GEO accession number GSE47879) we used a pseudoproband (permutation) approach to identify aberrantly methylated CpGs on autosomal chromosomes (loci on the X and Y chromosomes were excluded to avoid possible confounding effects of CpG methylation associated with X-inactivation). For this we successively assigned the patient and each normal control as a proband and determined the number of CpGs showing an aberrant methylation in comparison to the mean methylation levels of all other individuals. As a threshold for aberrant methylation we used delta β ≥ 0.3, where β is the ratio of methylated signal in relation to the overall signal of methylated and unmethylated with a scale from 0.0 as unmethylated to 1.0 as fully methylated [24]. CpGs passing the threshold (n=131; 87 CpGs associated with genes not known to be imprinted, 44 CpGs associated with known imprinted genes) are listed in Table S1. To characterize the methylation defect at genes of unknown imprinting status, all genes known to be imprinted at the time of analysis (February 2011; based on http://www.geneimprint.com and http://igc.otago.ac.nz) were excluded from further analysis. The 18 normal controls showed an average of about 15 CpGs associated with 15 genes with aberrant methylation (standard deviation (SD) 4.84). A similar number was found in the patient’s parents (mother: 15, father: 11). In contrast, 87 CpGs associated with 85 genes were observed in the patient (Figure 1), which is more than 14 times the SD (+14.9 SDs). The empirical p-value is 0.05 and the upper limit of the 95 % confidence interval is 0.176 (see Material and Methods).

For half of the aberrantly methylated loci (46 CpGs) the patient showed a hypomethylation as compared to the normal controls, whereas the other half were hypermethylated, supporting the hypothesis of a multilocus methylation defect rather than a multilocus hypomethylation. Out of the 85 candidate genes with unknown imprinting status, 19 were selected for in-depth methylation analyses by deep bisulfite sequencing (Table 1). We selected 16 hypomethylated and three hypermethylated genes. Eleven genes had an average methylation level (β-value) in the normal controls between or close to ~0.4 and 0.6 suggestive of a possible allele-specific methylation (Table 2). Other genes showed a high degree of hypo- or hypermethylation in the patient compared to the normal controls. Only loci were chosen where the CpG analysed on the 27k array could be investigated together with a neighbouring single nucleotide polymorphism (SNP), so that each amplicon included the CpG analysed on the array and a nearby SNP. Furthermore, at least two normal controls not investigated on the array had to be informative for these variants (Table S2) to enable the differentiation of the two parental alleles with the exception of *HKR1*, which was chosen as an example for high inter-individual variability. For all genes a literature research concerning previous methylation analyses was conducted at the time of analysis. A gene set enrichment analysis of the 85 candidate genes using GeneTrail (http://genetrail.bioinf.uni-sb.de/enrichment_analysis.php?js=1&cc=1) showed no significant enrichment for pathways or gene ontology categories after Bonferroni correction (data not shown).

**Table 1:** Genes investigated.

**Gene investigated**	**CpG (27k array ID)**	**CpG island**	**Genomic location**	**Patient shows**
*ACTN3*	cg08012287	no	chr11q13.1	hypomethylation
*AKR1C3*	cg19118077	no CpG Island	chr10p15.1	hypermethylation
*CCDC19*	cg02849695	CpG4 - CpG27	chr1q23.2	hypomethylation
*CHP2 (LOC63928)*	cg21745164	no	chr16p12.1	hypomethylation
*ECEL1^#^*	cg25431974 and cg02932167	yes	chr2q37.1	hypomethylation
*EDARADD*	cg09809672	no	chr1q43	hypermethylation
FAM50B*	cg01570885	yes	chr6p25.2	hypomethylation
*GAL3ST1*	cg09022808	no	chr22q12.2	hypomethylation
*HKR1*	cg12024906	CpG1 - CpG11 within	chr19q13.12	hypomethylation
*HOXB6*	cg16848873	no	chr17q21.32	hypomethylation
*KCNAB3*	cg14918082	no	chr17p13.1	hypomethylation
*KIF12*	cg17465304	yes	chr9q32	hypomethylation
*MAMDC2*	cg13870494	no	chr9q21.11	hypomethylation
*NAV1^#^*	cg14920846 and cg25167447	yes	chr1q32.1	hypomethylation
*POU3F1*	cg17791651	yes	chr1p34.3	hypomethylation
*SEC 31B (SEC 31L2)*	cg20831708	yes	chr10q24.31	hypomethylation
*TRPC3*	cg18474934	yes	chr4q27	hypomethylation
*TSPO (BZRP)*	cg00343092	yes	chr22q13.2	hypermethylation
*ZNF710*	cg01185080	yes	chr15q26.1	hypomethylation

The table lists the genes selected for in-depth methylation analyses together with the 27k array ID of the CpG. The third column shows whether the amplicon is located within a CpG island (UCSC browser; hg18). Only in the case of *AKR1C3* no CpG island was present. Column four gives the genomic location of the respective gene (UCSC browser; hg18). The methylation status of the patient as determined by 27k array analysis is given compared to the normal controls.

^#^ two CpGs affected; * recently shown to be imprinted [11,25]

**Table 2:** Results of the deep bisulfite sequencing methylation analyses.

	**Number of CpGs**	**Number of reads**					**Mean methylation level per amplicon (Roche/454 Genome Sequencer)**					**27k array methylation data**	
**Gene**	**per amplicon**	**Normal control**		**Normal control**		**Patient**	**Normal control**		**Normal control**		**Patient**	**NCs (average; range)**	**Patient**
***ACTN3***	5	2036	NC 5	2956	NC 6	1455	70.3	NC 5	73.9	NC 6	39.4	0.72; 0.65-0.81	0.32
***AKR1C3***	8	347	NC 3	282	NC 10	441	6.6	NC 3	1.5	NC 10	70.7	0.10; 0.07-0.18	0.75
***CCDC19***	27	446	NC 8	358	NC 9	188	28.1	NC 8	39.1	NC 9	19.9	0.46; 0.36-0.63	0.16
***CHP2***	9	301	NC 9	579	NC 13	567	73.0	NC 9	69.4	NC 13	49.9	0.63; 0.56-0.74	0.31
***ECEL1^#^***	31	2324	NC 3	1939	NC 14	1523	15.5	NC 3	14.8	NC 14	5.8	0.76; 0.56-0.86	0.28
***EDARADD***	7	910	NC 1	379	NC 9	787	16.2	NC 1	23.4	NC 9	33.0	0.48; 0.32-0.65	0.86
FAM50B*	17	669	NC 4	423	NC 15	656	52.7	NC 4	49.4^§^	NC 15	1.4	0.51; 0.46-0.55	0.19
***GAL3ST1***	5	2324	NC 17	3512	NC 18	1926	79.8	NC 17	75.8	NC 18	43.7	0.75; 0.66-0.83	0.42
***HKR1***	16	3 normal controls see Figure 6				550	3 normal controls see Figure 6				0.8	0.44; 0.23-0.67	0.02
***HOXB6***	11	620	NC 11	662	NC 13	393	53.3	NC 11	39.3	NC 13	22.2	0.57; 0.34-0.78	0.20
***KCNAB3***	5	803	NC 3	705	NC 5	2053	76.2	NC 3	76.7	NC 5	2.8	0.86; 0.69-0.94	0.06
***KIF12***	17	643	NC 5	1411	NC 21	664	67.1	NC 5	75.3	NC 21	51.3	0.85; 0.77-0.96	0.53
***MAMDC2***	7	393	NC 2	173	NC 5	266	44.9	NC 2	45.6	NC 5	12.1	0.77; 0.68-0.84	0.12
***NAV1^#^***	24	732	NC 4	1423	NC 6	544	49.8	NC 4	68.8	NC 6	13.6	0.55; 0.33-0.88	0.08
***POU3F1***	32	2278	NC 4	2020	NC 5	2084	30.5	NC 4	32.5	NC 5	8.8	0.61; 0.51-0.72	0.11
***SEC 31B***	28	1097	NC 6	1640	NC 8	1659	28.8	NC 6	29.2	NC 8	6.7	0.60; 0.36-0.76	0.14
***TRPC3***	24	8 normal controls see Figure 3				834	8 normal controls see Figure 3				8.5	0.62; 0.41-0.78	0.24
***TSPO***	21	2681	NC 18	1416	NC 19	1993	1.5	NC 18	0.7	NC 19	18.3	0.05; 0.02-0.12	0.38
***ZNF710***	40	441	NC 10	339	NC 12	468	63.6	NC 10	46.3	NC 12	29.2	0.64; 0.49-0.76	0.29

The table summarises the results obtained regarding methylation level [%] and number of reads. Additionally the methylation levels observed on the 27k array are given in β-values for comparison as well as the number of investigated CpGs per amplicon. In case of *HKR1* and *TRPC3* more than two normal controls were investigated; data are shown in the respective figures.

NC - normal control; ^#^ two CpGs affected - data for the CpG showing a greater difference in methylation are included; * recently shown to be imprinted [11,25]; ^§^ methylation level after correction for allelic imbalance in the obtained reads.

**Figure 2:**
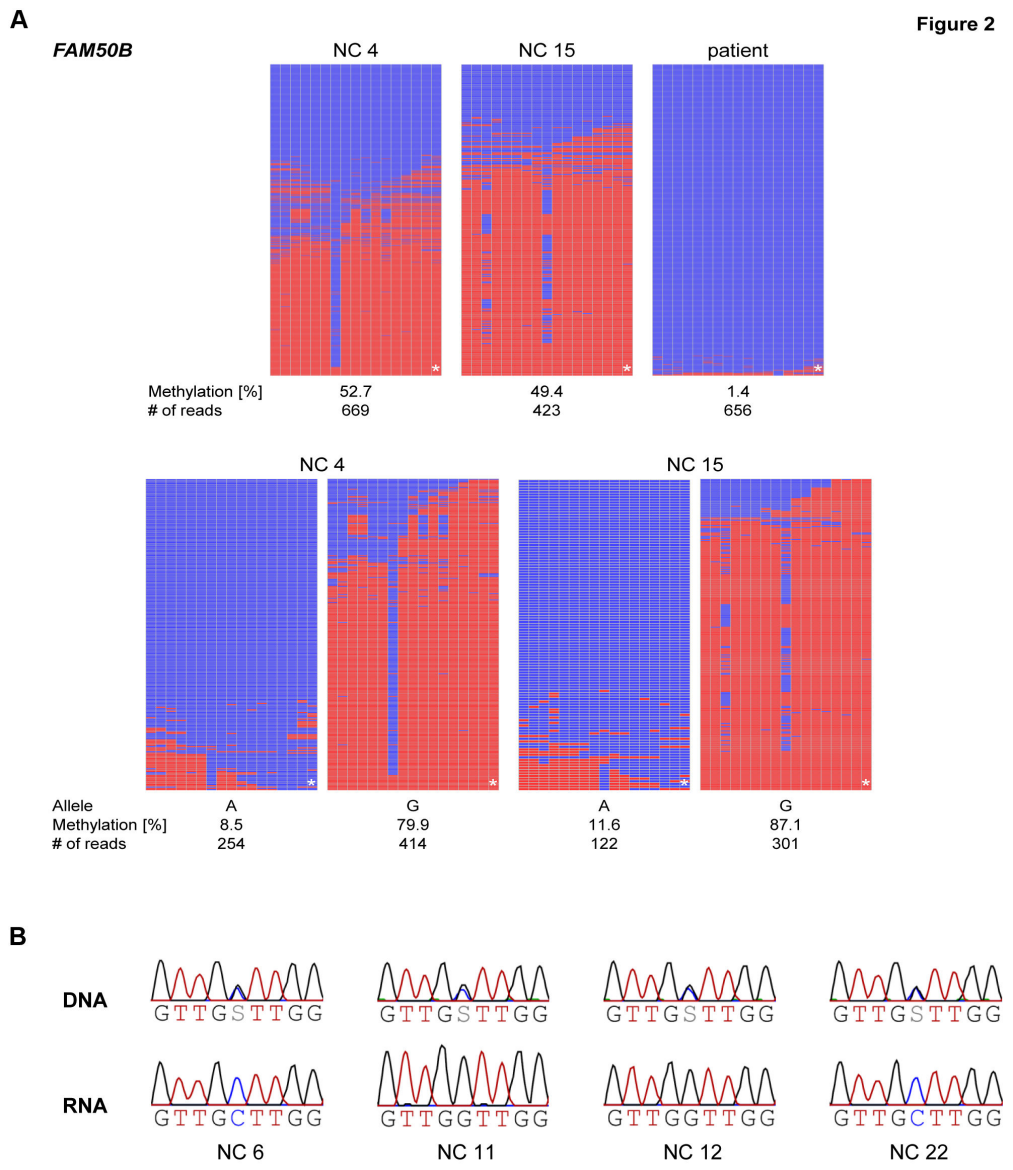
Results of methylation and expression analyses of *FAM50B* in blood. **a**) **Methylation analysis**. The figure shows the results of the methylation analyses by deep bisulfite sequencing of two normal controls (NC 4 and NC 15) and the patient (top). Heterozygosity for a SNP in both normal controls allowed to separate the alleles (bottom). Mean methylation levels, SNP allele and number of reads are given below each pattern. Lines represent reads; columns represent CpG dinucleotides; blue squares – unmethylated CpGs; red squares – methylated CpGs; white squares – missing sequence information; * – CpG investigated on the 27k array (CpG 17). **b**) Expression analysis. Expression analysis in four normal controls heterozygous for a SNP (rs6597007 C/G). For each normal control (NC) sequences obtained from peripheral blood DNA and RNA are shown. In the RNA only one allele is present, which indicates monoallelic expression.

Among the 19 candidate genes, only *FAM50B* had been proposed before to be possibly imprinted by Luedi et al. [6]. *FAM50B* is a retrotransposed gene located on human chromosome 6p25 and was recently reported to be imprinted [11,25]. They showed that *FAM50B* is subject to genomic imprinting in various tissues being methylated on the maternal allele and exhibiting expression from the paternal allele only. We studied the methylation status of *FAM50B* inside a CpG island in the promoter region in peripheral blood of two normal controls (Table 1). The detected degree of methylation was about 50 % confirming the 27k array data (average β in normal controls 0.51; Table 2). Separation of the two parental alleles showed an unmethylated and a methylated allele thus confirming allele-specific methylation (Table 3; Figure 2a; for SNP rs number see Table S2). Furthermore, expression analyses in blood displayed monoallelic expression indicating that *FAM50B* is subject to genomic imprinting in blood (Figure 2b), too.

**Table 3:** Results of the deep bisulfite sequencing methylation analyses after allele separation.

		**Mean methylation level per amplicon [%] (Roche/454 Genome Sequencer)**					**Number of sequence reads per amplicon**				
**Gene**	**Allele**	**Normal control**		**Normal control**		**Patient**	**Normal control**		**Normal control**		**Patient**
***ACTN3***	C	70.0	NC 5	74.3	NC 6	-	1060	NC 5	1428	NC 6	-
	G	70.5		73.6		-	964		1504		-
***AKR1C3***	A	6.3	NC 3	7.4	NC 10	-	129	NC 3	131	NC 10	-
	G	7.2		6.5		-	201		199		-
***CCDC19***	G	31.0	NC 8	36.9	NC 9	-	213	NC 8	149	NC 9	-
	C	25.5		42.2		-	233		182		-
***CHP2***	G	71.3	NC 9	73.8	NC 13	54.9	148	NC 9	278	NC 13	252
	C	74.6		64.9		45.3	149		291		302
***ECEL1***	A	16.3	NC 3	16.7	NC 14	-	1123	NC 3	1000	NC 14	-
	G	14.8		12.7		-	1185		920		-
***EDARADD***	A	16.2	NC 1	16.3	NC 9	-	443	NC 1	201	NC 9	-
	G	23.4		23.6		-	445		167		-
***FAM50B***	G	79.9	NC 4	87.1	NC 15	-	414	NC 4	301	NC 15	-
	A	8.5		11.6		-	254		122		-
***GAL3ST1***	A	80.4	NC 17	76.0	NC 18	-	1244	NC 17	1900	NC 18	-
	G	79.1		75.7		-	1071		1956		-
***HKR1***	A	10.5	NC 3	-	-	-	354	NC 3	-	-	-
	G	10.0		-		-	418		-		-
***HOXB6***	A	49.8	NC 11	36.7	NC 13	-	241	NC 11	303	NC 13	-
	G	55.5		41.5		-	379		359		-
***KCNAB3***	A	81.5	NC 3	81.8	NC 5	2.9	426	NC 3	365	NC 5	1059
	G	70.1		70.8		2.7	368		333		1010
***KIF12***	A	65.8	NC 5	74.7	NC 21	-	328	NC 5	708	NC 21	-
	G	68.6		75.9		-	314		703		-
***MAMDC2***	C	44.6	NC 2	40.9	NC 5	12.3	241	NC 2	110	NC 5	140
	A	44.3		50.6		11.9	149		61		126
***NAV1***	A	48.9	NC 4	71.1	NC 6	-	433	NC 4	539	NC 6	-
	G	51.4		67.3		-	295		866		-
***POU3F1***	A	31.3	NC 4	-	NC 5	-	697	NC 4	-	NC 5	-
	G	30.2		-		-	1579		-		-
***SEC 31B***	C	27.8	NC 6	27.2	NC 8	-	489	NC 6	689	NC 8	-
	G	28.6		30.0		-	600		932		-
***TRPC3***	C	see Figure 3				-	see Figure 3				-
	G					-					-
***TSPO***	G	1.0	NC 18	0.8	NC 19	22.4	1415	NC 18	645	NC 19	1066
	T	2.0		0.6		13.5	1252		769		922
***ZNF710***	G - G	70.3	NC 10	53.0	NC 12	-	142	NC 10	126	NC 12	-
	C - A	60.4		42.3		-	295		213		-

The table summarises methylation levels and number of reads after allele separation based on a present SNP (Table S2). The alleles are given as genomic sequence on the strand analysed. For *ZNF710* alleles for two SNPs are shown, G/C for NC 10 and G/A for NC 12. The numbers of reads for the two separated alleles may not add up to the read numbers before separation as some reads could not be assigned to the corresponding allele.

NC – normal control

By deep bisulfite sequencing, the patient had a methylation level of about 1.4 %, thus confirming the 27k array data (β = 0.20; Figure 2a) and showing an even more pronounced hypomethylation compared to the array. Separation of alleles was not possible due to the lack of an informative variant. The identification of this second imprinted gene - *FAM50B* - after the previously described *RB1* gene [23] emphasises the potential of this analysis to discover new imprinted genes. 

**Figure 3:**
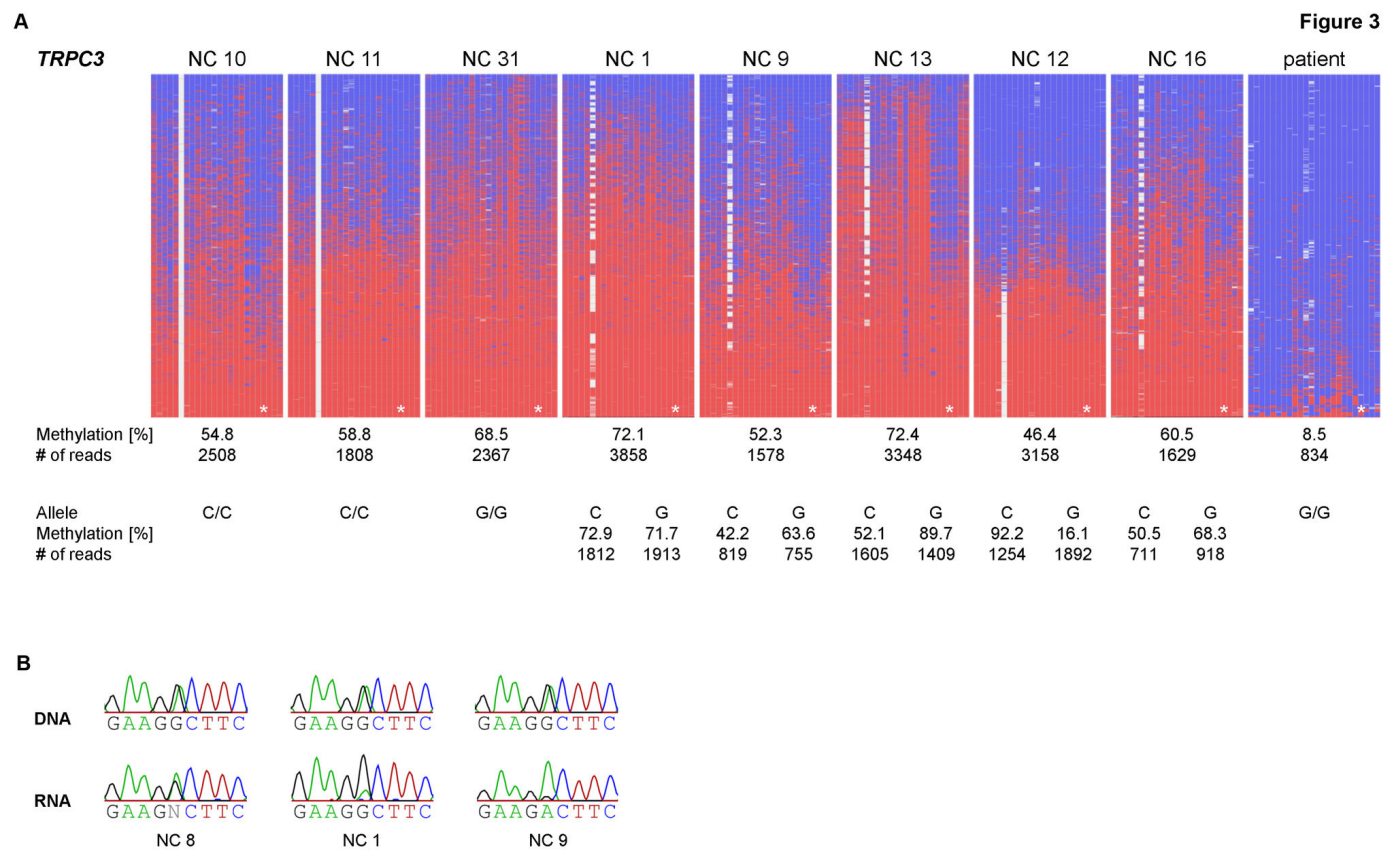
Results of methylation and expression analyses of *TRPC3* in blood. **a**) Methylation patterns of *TRPC3*. Results of the methylation analyses of 24 CpG dinucleotides obtained by deep bisulfite sequencing. Peripheral blood samples of eight normal controls (NC) and the patient were investigated. The present SNP at CpG 6 indicates the distribution of methylation across the two alleles. As it disrupts the CpG site 6, the white squares refer to the C allele, while filled squares refer to the G allele. Mean methylation levels, SNP allele and number of reads are given below each pattern. Lines represent reads; columns represent CpG dinucleotides; blue squares – unmethylated CpGs; red squares – methylated CpGs; white squares – missing sequence information, at CpG 6 due to a SNP (rs13121031) that disrupts the CpG dinucleotide; * – CpG investigated on the 27k array (CpG 21). **b**) Expression patterns of *TRPC3* in blood. Expression analyses in three normal controls heterozygous for a SNP (rs11732666 G/A). For each normal control (NC) sequences obtained from peripheral blood DNA and RNA are shown. Expression varies from biallelic to skewed.

Methylation analyses of *TRPC3* (*Transient receptor potential channel subfamily C, member 3*) were performed inside CpG island 55 located in the promoter region of transcript 2/isoform b (UCSC, hg18 uc003ief.1; NM_003305). The 27k array data showed a hypomethylation in the patient (β = 0.23), which we confirmed by deep bisulfite sequencing showing an even more pronounced hypomethylation (8 %). The methylation patterns of eight normal controls showed a high inter-individual variability in the degree of overall methylation ranging from 47 to 72 %, which again confirms the range observed in the 18 normal controls and the patient‘s parents on the 27k array (β = 0.41 - 0.78; Figure 3a, Figure S1 and Table 2). After allele separation in five normal controls heterozygous for a present SNP (rs13121031), the methylation observed showed a variable degree across both alleles (Figure 3a). Interestingly, in one individual (NC 12), a nearly allele-specific methylation pattern was present, showing methylation levels of 16 % and 92 % for the separated alleles, respectively, highlighting the high variability of the methylation (see Figure 3a). Investigation of three individuals homozygous for one allele showed degrees of methylation of 55 % and 59 % (C allele) and 69 % (G allele), respectively, which is in the range shown by the heterozygous individuals. 

*TRPC3* was also identified as a possible candidate gene for imprinting in a screen by Nakabayashi et al. [11]. Further analyses conducted by Martin-Trujillo et al. [26] via bisulfite PCR and cloning in four different tissues including leukocytes showed that the methylation seemed to be influenced by the genotype of the present SNP rs13121031 and that the major allele showed high methylation levels while the minor allele did not show methylation. We could not detect such an influence of the genotype of rs13121031 on the methylation by deep bisulfite sequencing in peripheral blood samples of eight homo- and heterozygous normal controls. In contrast to the previous report we investigated the methylation on the antisense strand from which *TRPC3* is transcribed. Therefore we cannot rule out a strand-specific difference in methylation although such reports were rare [27,28]. 

Methylation of the CpG Island 160 located in the promoter region of transcript a/isoform 1 (UCSC hg18, uc003ieg.1; NM_001130698) was investigated by Sanger sequencing of bisulfite treated DNA and showed only unmethylated CpG sites (data not shown) corresponding to results reported before [26].

Furthermore, expression analyses of *TRPC3* were conducted in RNA from peripheral blood of six informative normal controls for both isoforms together utilizing a SNP (rs11732666 in exon 8 of transcript 2/isoform b (UCSC hg18, uc003ief.1; NM_003305) and exon 9 of transcript 1/isoform a, respectively (UCSC hg18, uc003ieg.1; NM_001130698; Table S2). The expression patterns observed varied from biallelic to skewed expression in favour of one allele (Figure 3b). These results correspond to the findings of a variable methylation across both alleles and to the results by Martin-Trujillo et al. [26], where the expression in one fetal brain sample was investigated using the SNP rs13121031, which showed a skewed expression. 

**Figure 4:**
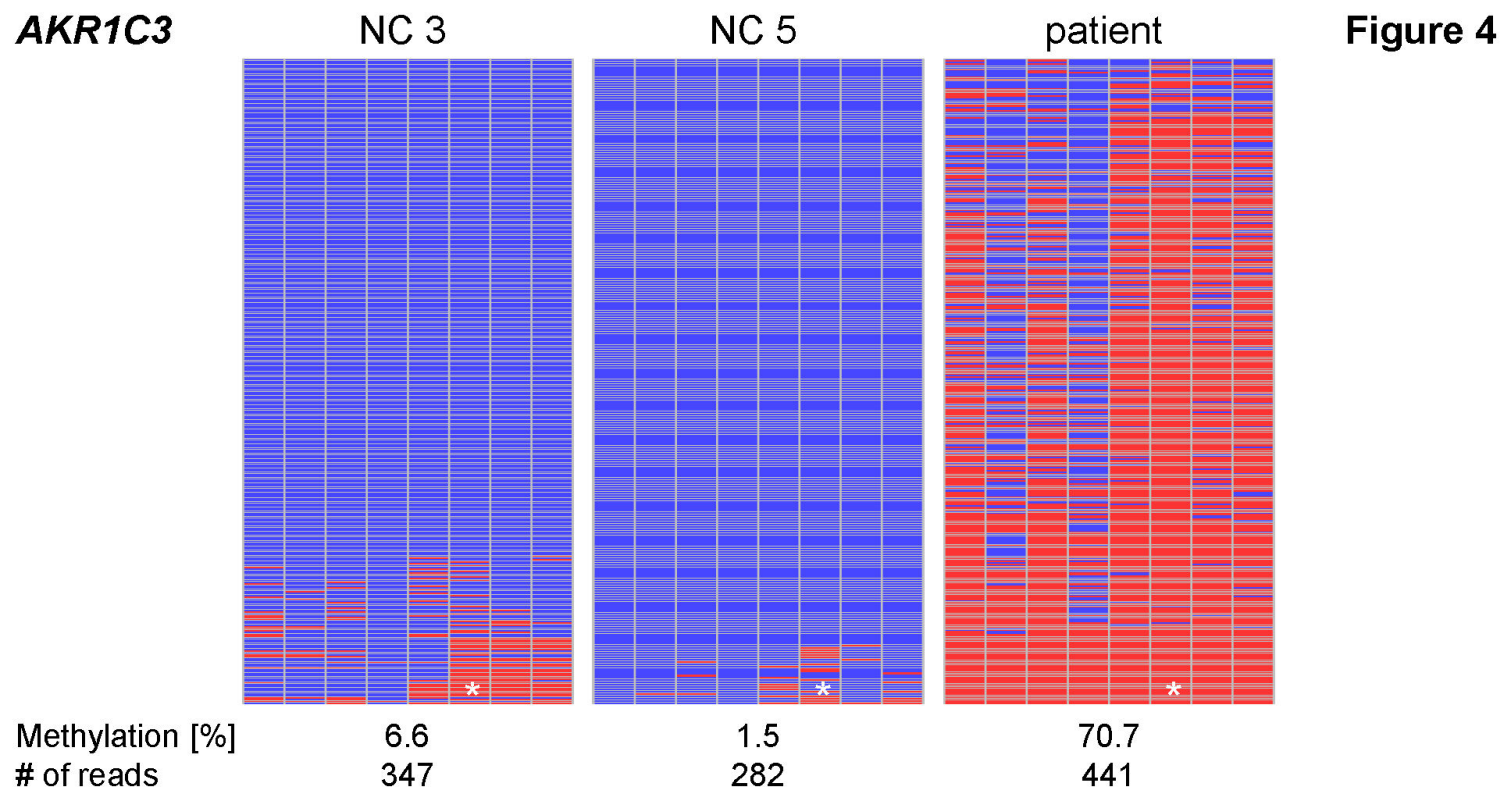
Methylation patterns of *AKR1C3*. For this locus the patient shows hypermethylation, whereas the two normal controls (NC) display very low levels of methylation. Mean methylation levels and number of reads are given below each pattern. Lines represent reads; columns represent CpG dinucleotides; blue squares – unmethylated CpGs; red squares – methylated CpGs; white squares – missing sequence information; * – CpG investigated on the 27k array (CpG 7).

As the array-based methylation data indicated that the patient has a more general methylation defect (Figure 1; Table S1) we additionally investigated three genes at which the patient showed a hypermethylation compared to the normal controls. One of these genes is *AKR1C3* (*aldo-keto reductase family 1, member C3*), where the patient showed a hypermethylation (β = 0.75) compared to the normal controls with an average β of 0.10 (range 0.07 - 0.18) in the 27k data By deep bisulfite sequencing a methylation level of 71 % was observed in the patient confirming the strong hypermethylation. In blood of two investigated normal controls a low level of methylation of 2 % and 7 % respectively was observed (Figure 4; Table 3). 

**Figure 5:**
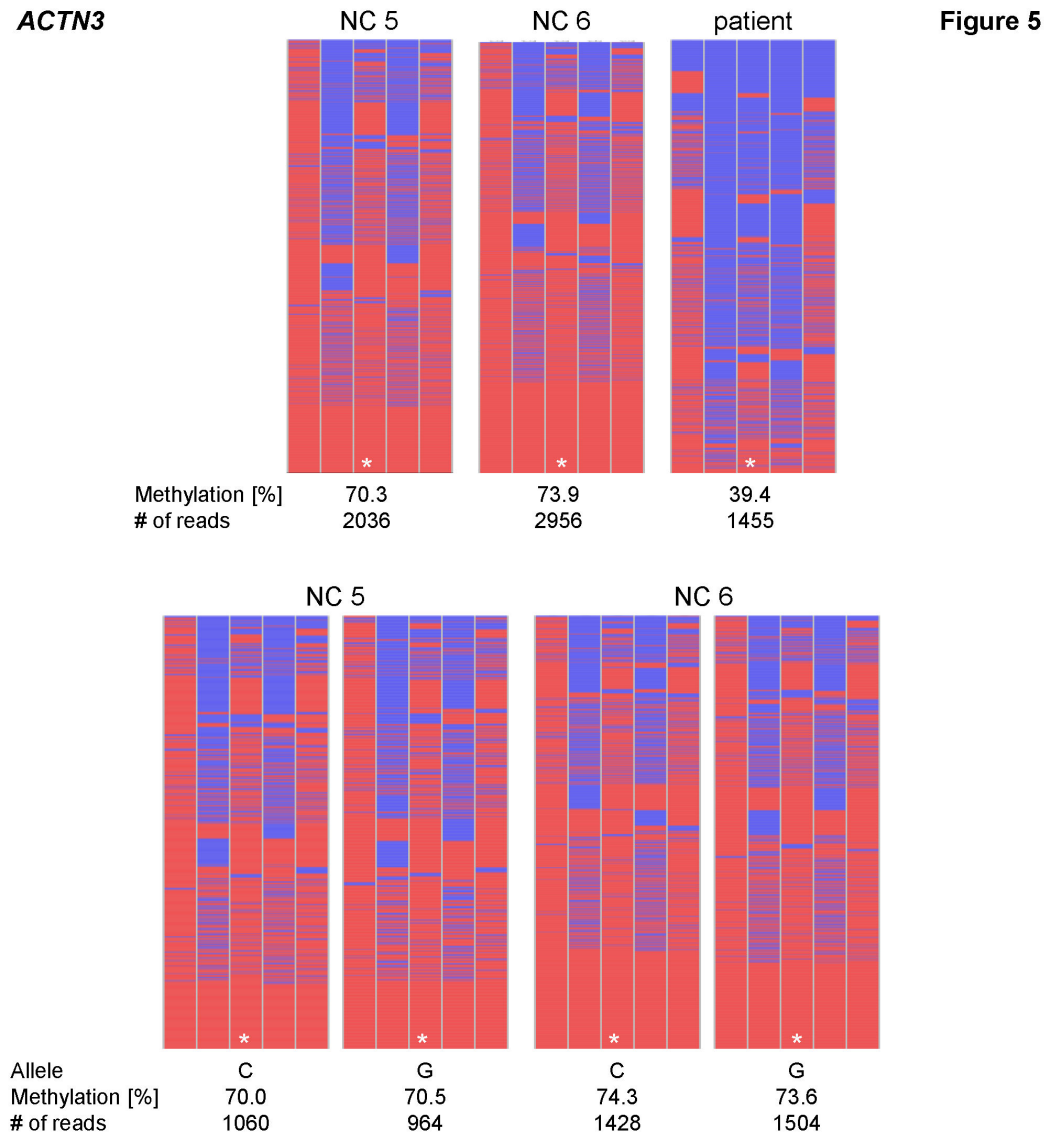
Methylation patterns of *ACTN3*. The two normal controls investigated display a high variability in methylation levels of adjacent CpGs. The same pattern is visible in the patient in a hypomethylated form. Mean methylation levels, SNP allele and number of reads are given below each pattern. Lines represent reads; columns represent CpG dinucleotides; blue squares – unmethylated CpGs; red squares – methylated CpGs; white squares – missing sequence information; * – CpG investigated on the 27k array (CpG 3).

The extensive methylation analyses conducted here also revealed and highlighted some interesting methylation patterns. One of these genes was *ACTN3* (*Actinin alpha 3*), which is located on chromosome 11q13.2. At this locus, a hypomethylation (β = 0.32) was detected in the patient compared to the normal controls (average β-value = 0.72) on the 27k array. The methylation values were verified by deep bisulfite sequencing, which showed 39 % methylation in the patient, whereas two normal controls showed methylation levels of 70 % and 74 % in blood, respectively. No significant differences in the methylation level or distribution across the alleles were present thus no allele-specificity. Interestingly, the degree of methylation between adjacent CpGs within the investigated area showed large differences. Whereas the CpGs 1, 3 and 5 of the amplicon exhibited methylation levels of about 80 - 90 %, the two CpGs in between (CpG 2 and 4) showed levels of only about 40 - 50 % (Figure 5 and Table 3). Such differences may be rare (as this was in one out of 19 genes) but should be borne in mind for analyses based on single CpGs only.

**Figure 6:**
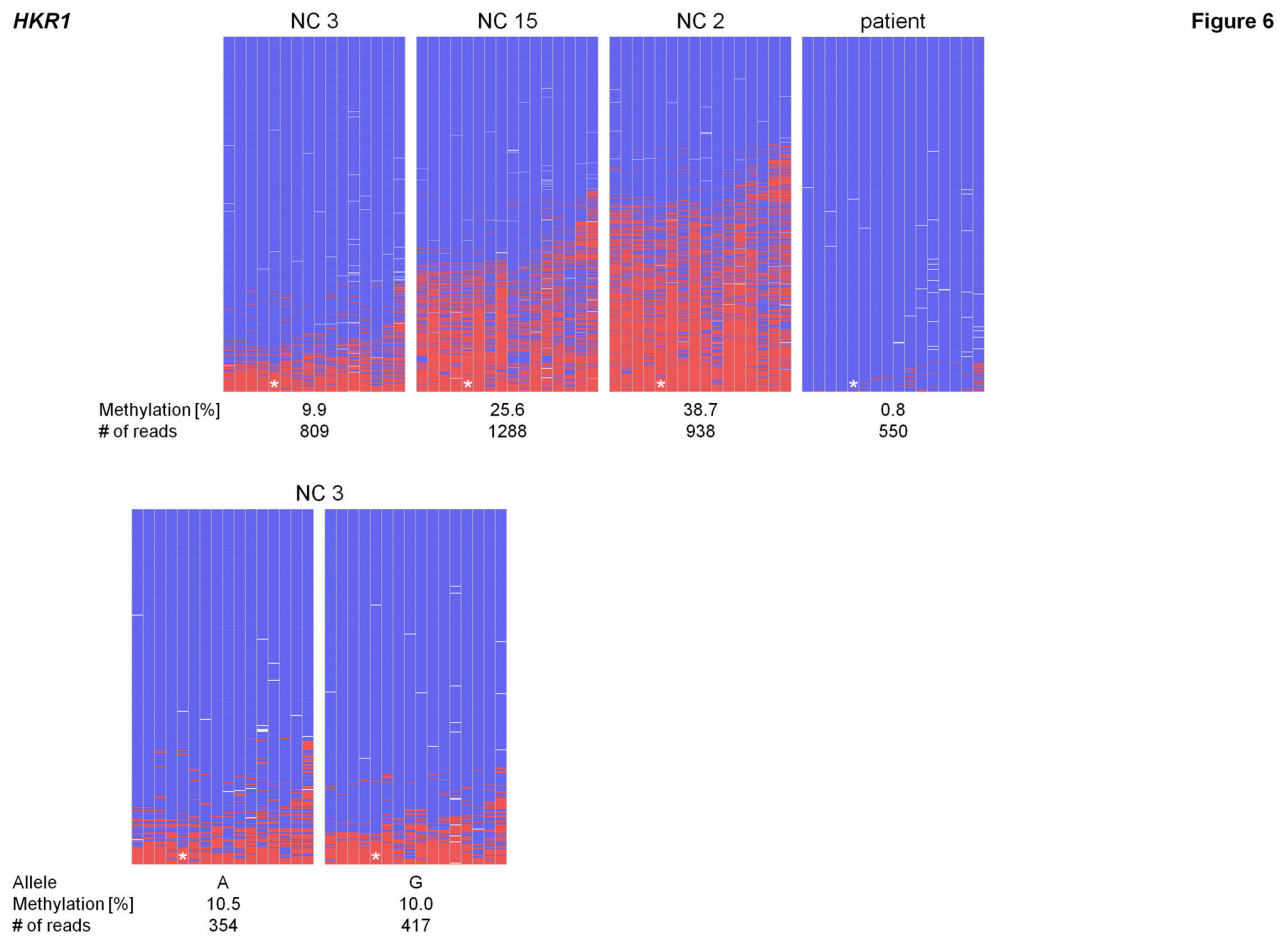
Methylation patterns of *HKR1*. Methylation patterns of three normal controls in blood displaying inter-individual variability. The methylation level in the patient is very low. In NC 3 the alleles could be separated (see lower part of the figure). Mean methylation levels, SNP allele and number of reads are given below each pattern. Lines represent reads; columns represent CpG dinucleotides; blue squares – unmethylated CpGs; red squares – methylated CpGs; white squares – missing sequence information; * – CpG investigated on the 27k array (CpG 5).

Another interesting example is *HKR1* (*Krueppel-related zinc finger protein 1*) on chromosome 19q13.13. Here the array data showed a broad range of methylation in the normal controls between β 0.23 and 0.67 while the patient showed a nearly complete loss of methylation with β of only 0.02. Analyses performed on the Roche/454 Genome Sequencer confirmed the unmethylated state in the patient, obtaining nearly exclusively unmethylated reads, while residual methylation was present at a few single CpGs only (Figure 6). Due to the variability in methylation seen on the array, three normal controls were investigated showing methylation levels of 9.9, 25.6 and 38.7 % respectively. Allele-separation could only be performed in one of these normal controls (NC 3) due to the absence of an informative variant in the others (Table S2). The methylation was evenly distributed across both alleles and thus no allele-specific pattern was present (Figure 6 and Table 3). These results demonstrate that there can be large inter-individual differences [29-31].

For the candidate genes investigated in more detail by deep bisulfite sequencing we could not detect allele-specific methylation or imprinting except for the previously described *FAM50B* [11,25]. Analyses after allele-separation did not reveal significant differences in level or distribution of methylation between the two parental alleles. This is also the case in the four genes *CHP2, KCNAB3, MAMDC2* and *TSPO* (Figure S2), where allele-separation was possible in the patient. This emphasises that the methylation defect in the patient affects both parental alleles. Previous reports suggested that mutations in *NLRP7* lead to a failure in the establishment of the maternal imprint in the female germline or in the postzygotic maintenance, as the investigated maternally methylated imprinted genes showed - if affected - a hypomethylation while the investigated paternally imprinted genes exhibited a hypermethylation [12,13,20,32,33]. Our and previous data ([23]; Caliebe et al. in preparation) suggest that not only the establishment and maintenance of the maternal methylation patterns can be affected, but also the methylation at imprinted and non-imprinted loci on the paternal allele . The results obtained for four genes (*CHP2, KCNAB3, MAMDC2* and *TSPO*) where we could separate the alleles in the patient showed hypomethylation of both parental alleles. This supports the hypothesis that methylation maintenance is affected and that this failure in methylation maintenance also affects non-imprinted loci. 

Most of the genes investigated (11 out of 19) showed an average level of methylation between 40 - 60 % in normal controls, as would be the case for an imprinted locus. We could verify allele-specific DNA methylation and expression of *FAM50B* in blood, but could not detect any other imprinted gene by this approach. 

The investigated regions were located within CpG islands in the majority of genes (11 out of 19 based on UCSC, hg18). Although most CGIs in promoter regions are unmethylated to allow transcription, alterations have frequently been reported in cancer [1,2,34]. The genes investigated here in more detail display a broad variety of different methylation levels within CGIs and in the 5' regions of genes and may thus influence transcription and contribute to phenotypic variance as has recently been discussed [35].

Only *TSPO* and *AKR1C3* showed very low methylation levels in the normal controls, which is in accordance with the frequently observed unmethylated state of CGIs in promoter regions. Although no CGI is present in *AKR1C3* (according to the parameters used by UCSC hg18 for CGI detection [36]) the investigated region overlaps with an alternative transcription start site of known isoforms (UCSC). 

As methylation can be influenced by or even depend on different *cis*- and trans-acting factors such as a present genotype (for review see 37) we analysed our data regarding the influence of the investigated SNPs. Evidence for a genotype dependent methylation based on the SNPs present in the investigated amplicons could not be observed (Table 3). Additionally, the normal controls investigated alongside the patient have been chosen to represent both sexes when applicable to be able to detect possible sex-specific differences in methylation as have been described before [38,39]. At the loci investigated such an effect was not present neither before nor after separation of the two alleles. 

The patient displayed moderate to severe hypo- and also hypermethylation at the investigated loci with differences in the degree of methylation ranging from about 10 - 60 % as compared to the matching normal controls. Why different loci are affected to a varying extend by methylation changes needs to be elucidated. A stochastic effect or underlying sequence differences including binding sites may be possible contributors. Similar effects have been observed e.g. for mutations in *ZFP57* or *TRIM28* [40-42].

In summary, our data show that some loci have considerable variation in DNA methylation and that the patient has aberrant methylation at imprinted and apparently non-imprinted loci. 

## Materials and Methods

### Ethics statement

Blood samples were obtained after written informed consent. For the patient we obtained written informed consent from his parents. All participants and the patient’s parents provided written informed consent for publication of case details. Control blood samples from blood donors were anonymised. The study was approved by the ethics committees of the Universities Kiel and Duisburg-Essen.

### Isolation of DNA and RNA

DNA and RNA were isolated from blood with the FlexiGene DNA Kit (Qiagen, Hilden, Germany) according to the manufacturer’s manual. RNA was extracted using the RNeasy Blood Kit (Qiagen, Hilden, Germany).

### High-throughput methylation proﬁling

DNA from peripheral blood of the patient, his parents and 18 unrelated healthy controls (a-c, e-q, s, t) was isolated using standard methods and analysed on Illumina Infinium HumanMethylation27 BeadChips, which quantitatively measure the methylation level at 27,578 single CpG sites. DNA bisulfite conversion was performed by applying the Zymo EZ DNA Methylation Kit (Zymo Research, Orange, CA, USA) according to the manufacturer’s protocol but with the modiﬁcations described in the Inﬁnium Assay Methylation Protocol Guide (Illumina Inc, San Diego, CA, USA). Subsequent analysis steps were performed according to the Inﬁnium II Assay Lab Setup and Procedures and the Inﬁnium Assay Methylation (http://www.illumina.com/technology/inﬁnium_methylation_assay.ilmn; accessed August 2009) Protocol Guide (http://www.illumina.com/products/inﬁnium_ humanmethylation27_beadchip_kits.ilmn#documentation; accessed August 2009) (Illumina Inc). Subsequently, DNA samples were hybridised to the HumanMethylation27 DNA Analysis BeadChip (http://www.illumina.com/products/inﬁnium_humanmethylation27_ beadchip_leits.ilmn; accessed August 2009) (Illumina Inc). Data analysis was performed using BeadStudio software (default settings; Illumina Inc). Technical replicates of DNA samples from the patient and his parents showed a high degree of correlation (analysis of the patient’s DNA is shown in Figure S3). For the pseudoproband approach, values were averaged.

### Statistical analyses

In the pseudoproband (permutation) approach each of the 18 unrelated normal controls who did not show aberrant methylation for more than one CpG associated with an imprinted locus and the patient were assigned separately as a pseudoproband to investigate the number of CpGs (genes) showing aberrant methylation. For this, the methylation level (β-value) of each pseudoproband at the investigated CpGs was compared to the average of the methylation levels of all other investigated individuals. If the methylation showed a difference greater than or equal to the set threshold of 0.3 β-values, the methylation at those CpGs was considered as aberrant. In an additional step, the pseudoproband approach was repeated including the patient’s parents.

To determine whether the relatively high number of CpGs with aberrant methylation observed in the patient compared to the normal controls could have occurred by chance, we calculated the empirical p-value from Monte Carlo procedures as described by Davison and Hinkley [43,44]. The empirical p-value (p_emp_) of an observation is the number of permutations that show a score higher than or equal to the actually observed score (r), divided by the number of all permutations (n) (p_emp_=r/n). After correction for a low number of r, the p-value is determined as p_emp_=(r+1)/(n+1). As none of the normal controls showed more CpGs with aberrant methylation in the permutations than the 87 observed in the patient, p_emp_ is 0+1/19+1 = 0.05. The upper limit of the 95 % confidence interval was determined as described by Clopper and Pearson [45]. Mean values and standard deviations (SD) were determined by standard procedures.

### Genotyping

For allele-specific analyses, normal individuals informative for SNPs in the genes investigated were identified. PCR was conducted using standard protocols and the products were sequenced using the BigDye Terminator v1.1 Cycle Sequencing Kit on an ABI-3100 automatic capillary genetic analyser (Applied Biosystems, Fostercity, CA, USA; primer sequences Table S3). Sequencing Analysis (Applied Biosystems, Fostercity, CA, USA) and Geneious (Biomatters, Auckland, New Zealand) were used for analyses.

### Deep bisulfite sequencing using the Roche/454 Genome Sequencer

Bisulfite treatment of genomic DNA was carried out as described before [46] or with the EZ DNA Methylation-Gold Kit (Zymo Research Europe, Freiberg, Germany) according to the manufacturer’s protocol. Locus-specific bisulfite amplicon libraries were generated with tagged primers using the Qiagen HotStarTaq Master Mix Kit (Qiagen, Hilden, Germany; primer sequences Table S3). Sample-specific barcode sequences (MIDs = multiplex identifiers) and universal linker tags (454 adaptor sequences, A- or B-primer and key) were added in a second PCR. Sample preparation and sequencing on the Roche/454 GS junior were carried out as described elsewhere [47]. Briefly, amplicons were purified using the Agencourt AMPure XP Beads (Beckman Coulter, Krefeld, Germany) system according to the protocol recommended by Roche, then quantitated with the NanoDrop ND-1000 Spectrophotometer (ThermoScientific, Wilmington, USA). The libraries were diluted, pooled and clonally amplified in an emulsion PCR (emPCR). Sequencing was conducted on the Roche/454 GS junior system according to the manufacturer’s protocol (Roche emPCR Amplification Method Manual - Lib-A and Roche Sequencing Method Manual). 

For data analyses special filter settings were applied to increase the yield of reads. The default “filterOnlyAmplicons” quality filter that is applied when using the “full processing for amplicons” option in the Genome Sequencer application was modified to increase the yield of reads from sequencing runs with bisulfite-treated DNA. The following two parameters were adapted: First the <doValleyFilterTrimBack> was set to true (the default being false). This option enables or disables the trim back filter. The default “false” setting exclusively accepts full-length reads (i.e., a read with low quality at its end would be discarded instead of trimmed), whereas the “true” setting allows trimmed sequences. Secondly, the <vfBadFlowThreshold> was set to “10” (the default being "4"). This parameter controls the number of bad flows tolerated before the read is discarded, reads with less bad flows are trimmed. Increasing this value increases the number of wells that pass the quality filter while reducing overall read quality.

Sequence analyses were conducted with the BiQAnalyzer HT after separation of reads per MID with the Geneious software (Biomatters, Auckland, New Zealand) [48]. The mean methylation over all CpGs and reads per sample and per amplicon are given. At least 150 reads per sample (average 1275) were analysed, having a conversion rate above 98 %. 

### Expression analyses

Reverse transcriptase (RT)-PCR was conducted using standard protocols. When necessary, RNA was prior treated with RQ1 DNase (Promega, Mannheim, Germany) to remove all traces of DNA. The subsequent PCR was performed with the AmpliTaq Gold (Applied Biosystems, Fostercity, CA, USA; primer sequences Table S3) and a touchdown PCR modified from Zeschnigk et al. [49] as described previously [50]. To exclude a contamination with genomic DNA, amplification of an RNA specific product for the β-actin gene was carried out [46]. The PCR products were either gel-purified using the Qiagen MinEute Gel Extraction kit (Qiagen, Hilden, Germany) or by use of ExoSap-It (USB, Ohio, USA, see manual). Sequencing and sequence analysis were performed as described above. 

## Supporting Information

Figure S1 **Methylation patterns of TRPC3 separated by alleles.** For normal controls informative for a SNP (rs13121031) the methylation patterns are shown for each allele separately. As the present SNP disrupts the CpG site 6, the white squares refer to the C allele, while filled squares refer to the G allele.
Mean methylation levels, SNP allele and number of reads are given below each pattern. Lines represent reads; columns represent CpG dinucleotides; blue squares – unmethylated CpGs; red squares – methylated CpGs; white squares – missing sequence information, at CpG 6 due to a SNP (rs13121031) that disrupts the CpG dinucleotide; * – CpG investigated on the 27k array (CpG 21).
(TIFF)

Figure S2 **Methylation patterns of a) *CHP2*, *b*) *KCNAB3*, c) *MAMDC2* and d) *TSPO*.** For each gene the methylation patterns of two normal controls and the patient are depicted before and after allele separation.
Mean methylation levels, SNP allele and number of reads are given below each pattern. Lines represent reads; columns represent CpG dinucleotides; blue squares – unmethylated CpGs; red squares- methylated CpGs; white squares – missing sequence information in case of *CHP2* due to a present SNP affecting a CpG dinucleotide; * – CpG investigated on the 27k array (*CHP2*: CpG 6, *KCNAB3*: CpG 3, *MAMDC2*: CpG 2 and *TSPO*: CpG 10).
(PDF)

Figure S3 **Scatterplot of two independent hybridisations of the patient’s DNA on the 27k array.** Note the high Pearson and Spearman correlation coefficient.
(PDF)

Table S1 **CpGs displaying aberrant methylation in the patient.**
(XLS)

Table S2 **Identifiers for SNPs used for allele-separation.**
(XLS)

Table S3 **Primer sequences.**
(XLS)
