# Correction: Deep Bisulfite Sequencing of Aberrantly Methylated Loci in a Patient with Multiple Methylation Defects

**DOI:** 10.1371/annotation/8a05c514-48b6-4dd9-b322-273b575cbd95

**Published:** 2013-12-05

**Authors:** Jasmin Beygo, Ole Ammerpohl, Daniela Gritzan, Melanie Heitmann, Katrin Rademacher, Julia Richter, Almuth Caliebe, Reiner Siebert, Bernhard Horsthemke, Karin Buiting

This paper was republished on December 4 due to a Table being erroneously incorporated into the text. Please download the corrected version. 

