# Supplementary figures and images for: Deep Bisulfite Sequencing of Aberrantly Methylated Loci in a Patient with Multiple Methylation Defects
Source: PLoS One. 2013 Oct 9;8(10):e76953. doi: 10.1371/journal.pone.0076953 (PMC3793946; doi:10.1371/journal.pone.0076953)

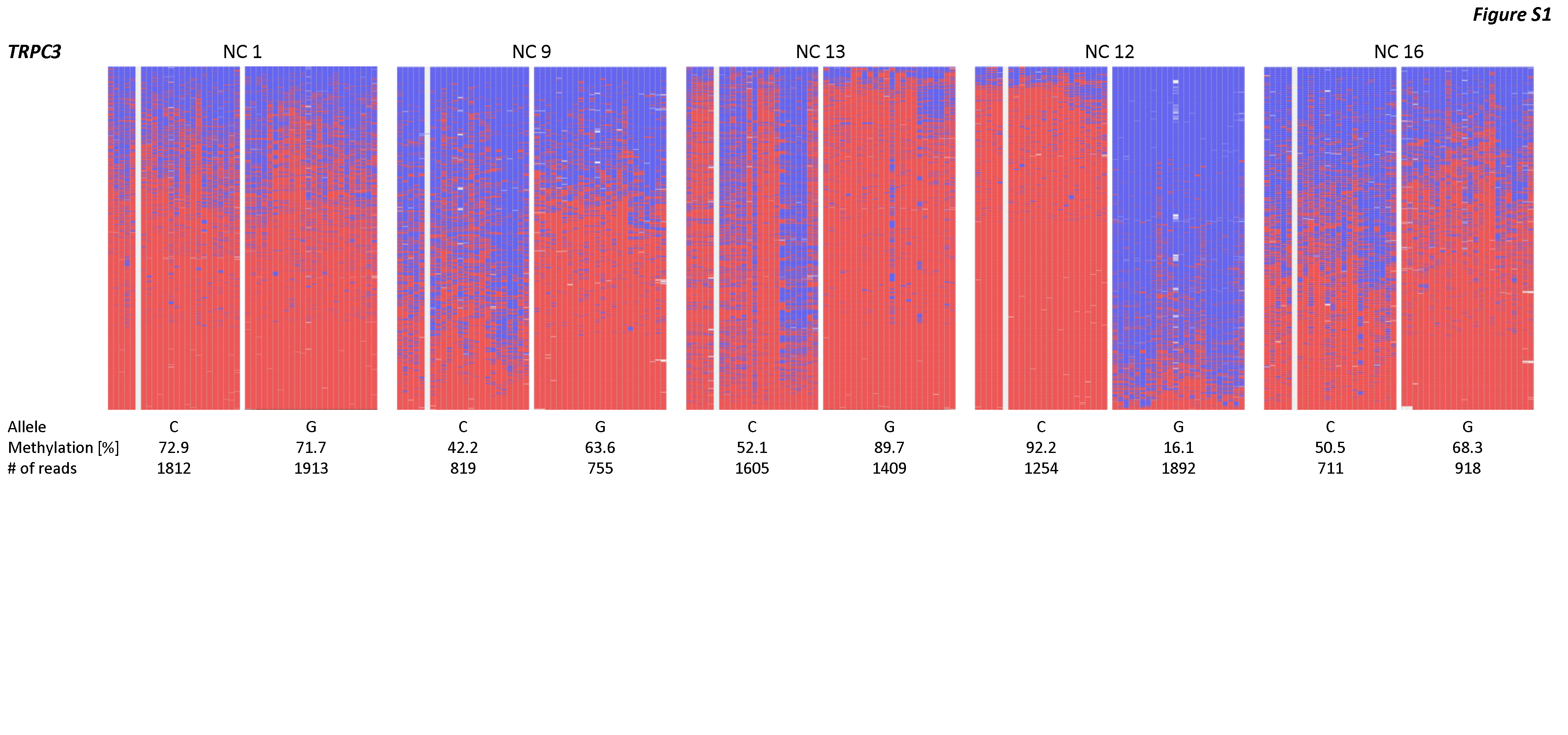

Supplement: Figure S1 — Methylation patterns of TRPC3 separated by alleles. For normal controls informative for a SNP (rs13121031) the methylation patterns are shown for each allele separately. As the present SNP disrupts the CpG site 6, the white squares refer to the C allele, while filled squares refer to the G allele. Mean methylation levels, SNP allele and number of reads are given below each pattern. Lines represent reads; columns represent CpG dinucleotides; blue squares – unmethylated CpGs; red squares – methylated CpGs; white squares – missing sequence information, at CpG 6 due to a SNP (rs13121031) that disrupts the CpG dinucleotide; * – CpG investigated on the 27k array (CpG 21). (TIFF) [file pone.0076953.s001.tiff]
