# Supplementary material for: Deep Bisulfite Sequencing of Aberrantly Methylated Loci in a Patient with Multiple Methylation Defects
Source: PLoS One. 2013 Oct 9;8(10):e76953. doi: 10.1371/journal.pone.0076953 (PMC3793946; doi:10.1371/journal.pone.0076953)

Figure S2

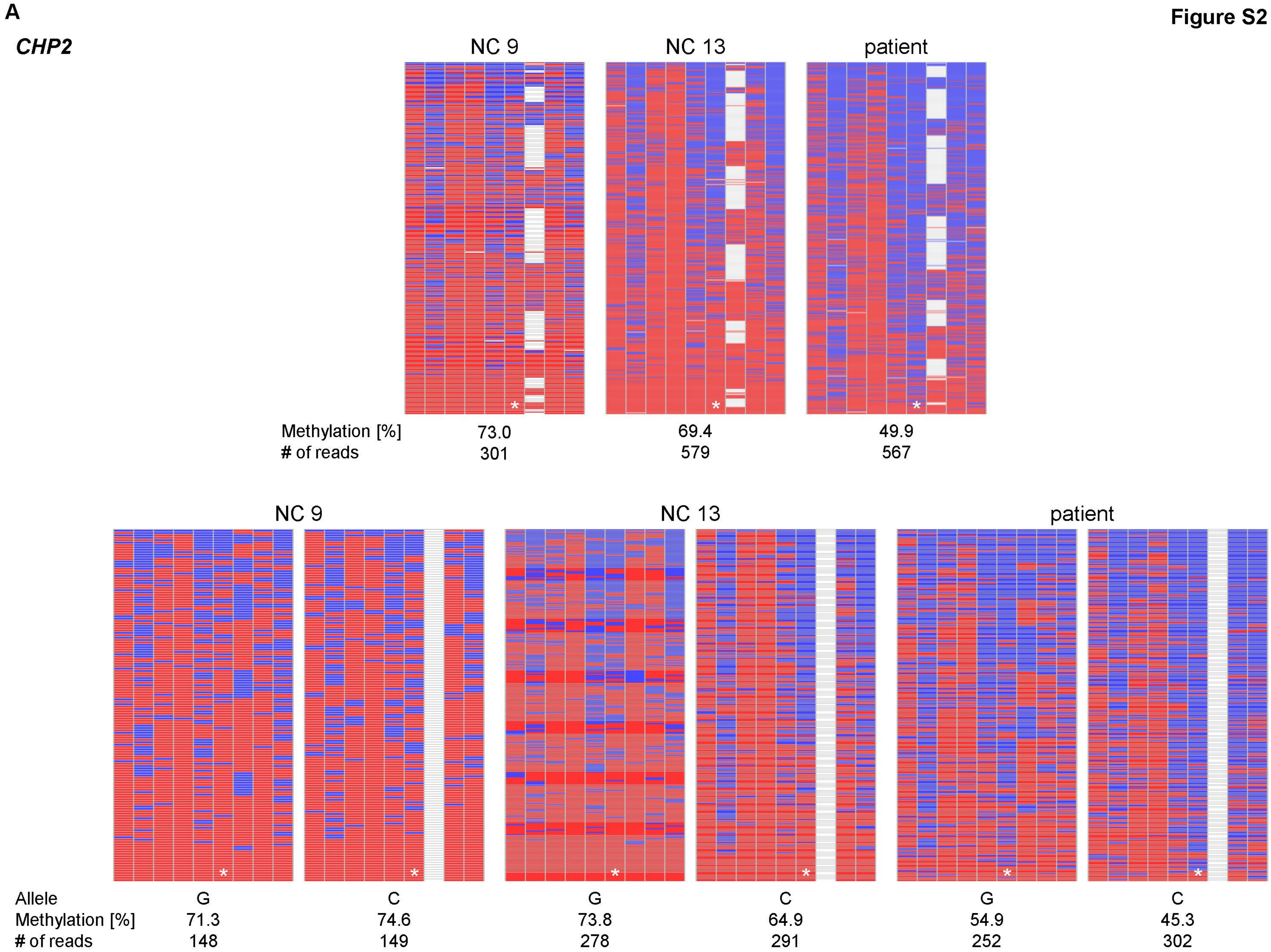
A
CHP2
NC 9
NC 13
patient
*
Methylation [%]
73.0
69.4
49.9
# of reads
301
579
567
NC 9
NC 13
patient
Allele
G
C
G
C
G
C
Methylation [%]
71.3
74.6
73.8
64.9
54.9
45.3
# of reads
148
149
278
291
252
302

B

Figure S2

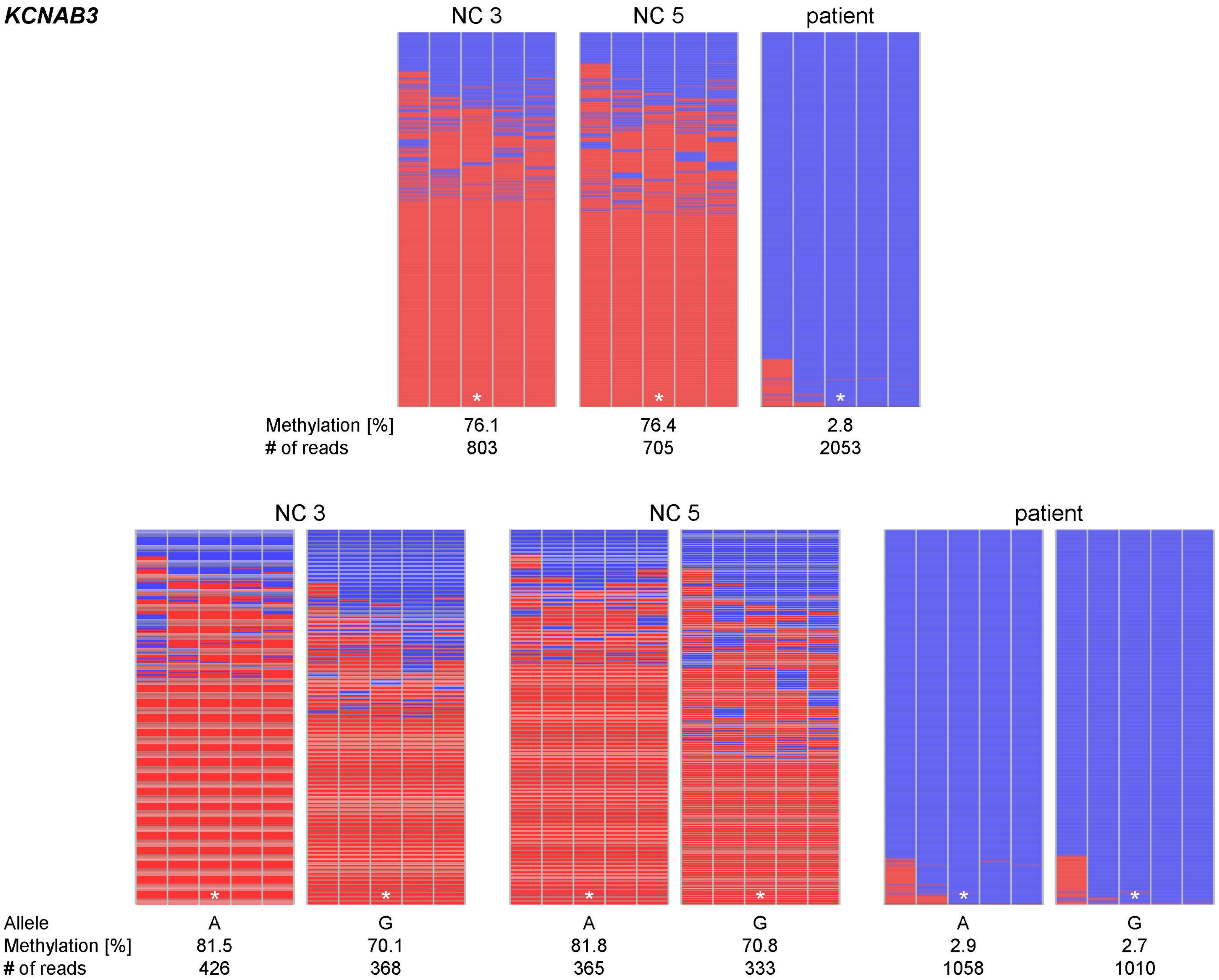
KCNAB3
NC 3
NC 5
patient
*
Methylation [%]
76.1
76.4
2.8
# of reads
803
705
2053
NC 3
NC 5
patient
Allele
A
G
A
G
A
G
Methylation [%]
81.5
70.1
81.8
70.8
2.9
2.7
# of reads
426
368
365
333
1058
1010

Figure S2

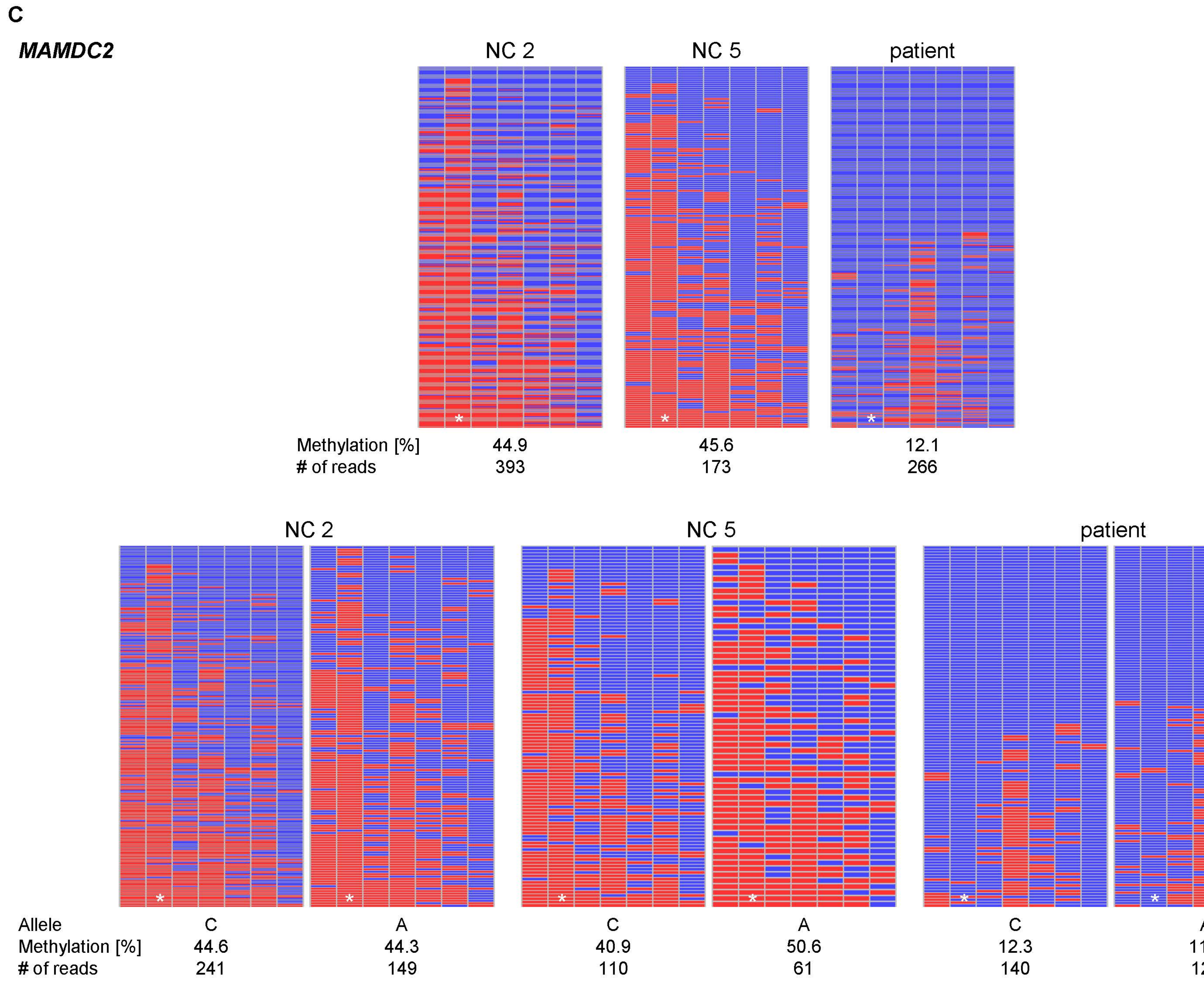
C
MAMDC2
NC 2
NC 5
patient
*
Methylation [%] 44.9 45.6 12.1
# of reads 393 173 266
NC 2
NC 5
patient
Allele C A C A C A
Methylation [%] 44.6 44.3 40.9 50.6 12.3 11.9
# of reads 241 149 110 61 140 126

D

Figure S2

TSPO

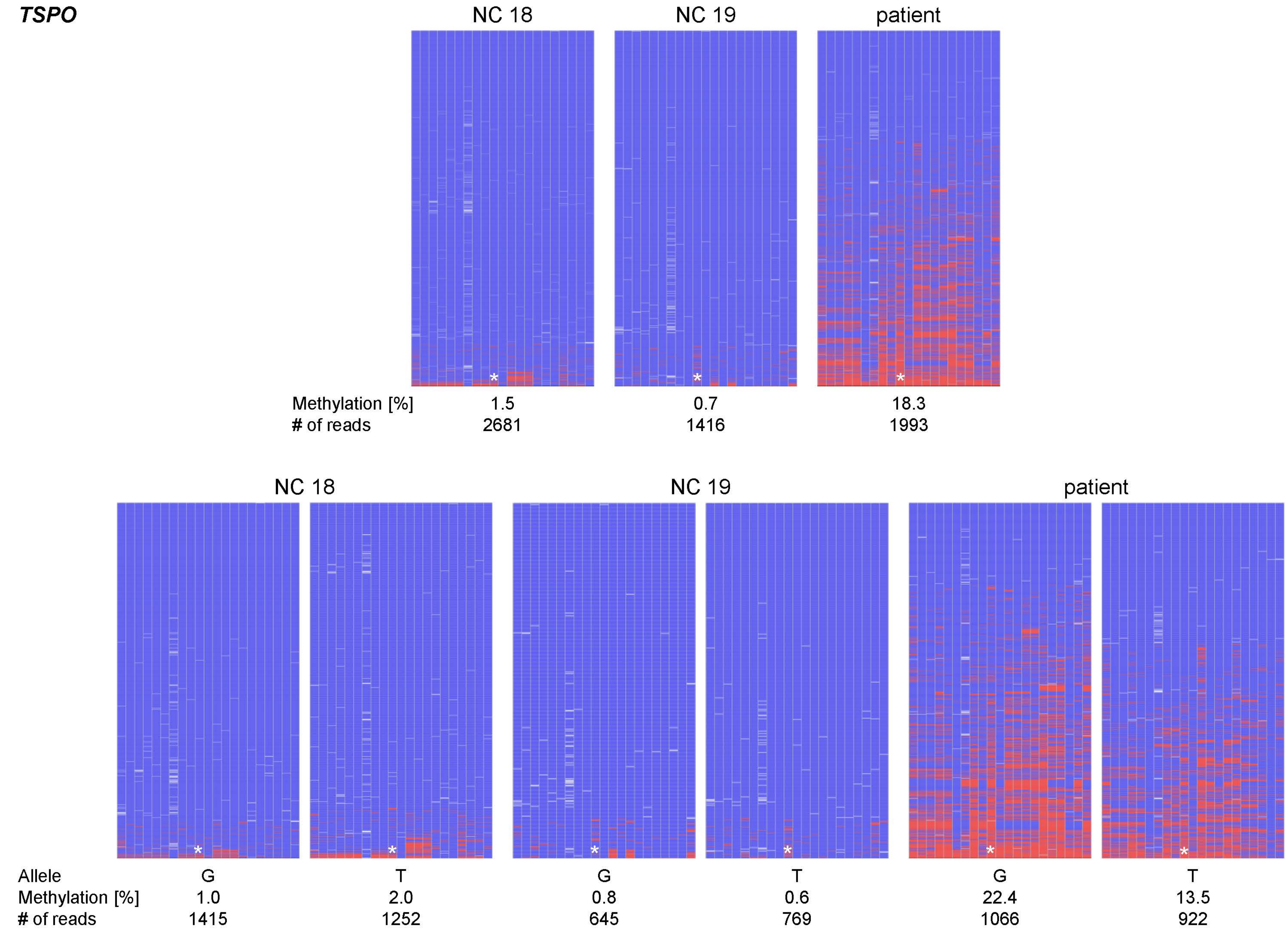

Supplement: Figure S2 — Methylation patterns of a) CHP2, b) KCNAB3, c) MAMDC2 and d) TSPO. For each gene the methylation patterns of two normal controls and the patient are depicted before and after allele separation. Mean methylation levels, SNP allele and number of reads are given below each pattern. Lines represent reads; columns represent CpG dinucleotides; blue squares – unmethylated CpGs; red squares- methylated CpGs; white squares – missing sequence information in case of CHP2 due to a present SNP affecting a CpG dinucleotide; * – CpG investigated on the 27k array (CHP2: CpG 6, KCNAB3: CpG 3, MAMDC2: CpG 2 and TSPO: CpG 10). (PDF) [file pone.0076953.s002.pdf]
