# Supplementary material for: Deep Bisulfite Sequencing of Aberrantly Methylated Loci in a Patient with Multiple Methylation Defects
Source: PLoS One. 2013 Oct 9;8(10):e76953. doi: 10.1371/journal.pone.0076953 (PMC3793946; doi:10.1371/journal.pone.0076953)
Supplement: Figure S3 — Scatterplot of two independent hybridisations of the patient’s DNA on the 27k array. Note the high Pearson and Spearman correlation coefficient. (PDF) [file pone.0076953.s003.pdf]

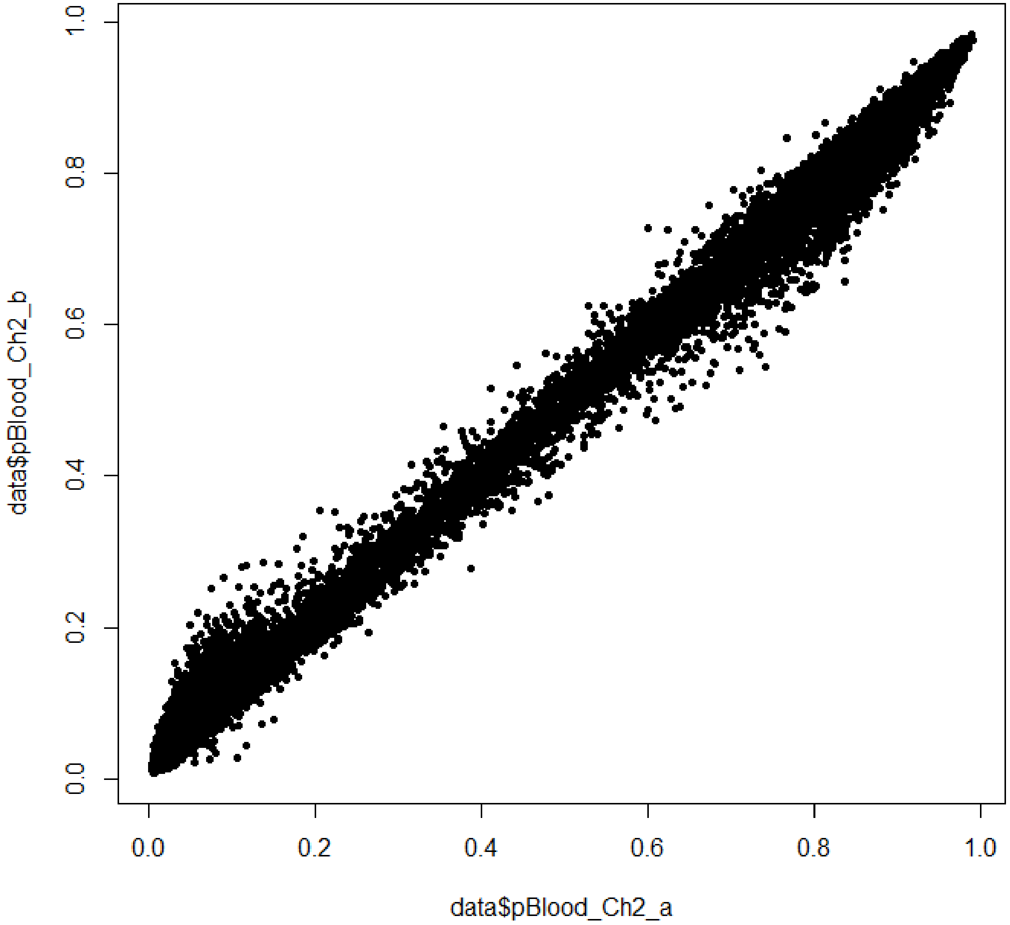

2 independent hybridisations
(peripheral blood, patient)

<u>coefficient of determination:</u>

Pearson: 0.9980

Spearman: 0.9718
